# Bromide Potassium to Suppurating Tumors

**Published:** 1875-04

**Authors:** 


Bromide of Potassium as a Caustic, Anæsthetic and Curative Application to Malignant, Suppurating Tumors.—Peyrand (*Il Raccoglitore Med.*) had a cancroid of the face, which produced excruciating pains, and which could not be extirpated. He determined to sprinkle the surface with finely pulverized bromide of potassium, after doing which the pains diminished in intensity and the growth of the fungous granulations was checked. This treatment was continued for four weeks with excellent results, and during this time the tumor entirely subsided. In this case the bromide exerted an anæsthetic influence on the terminal fibres of the sensitive nerves, diminishing the pain, and acted also through its vaso-motor peculiarity, by contracting the vessels of the tumor, and thus diminishing its volume. It acted finally as a caustic, and destroyed the superficial tissue; upon this point Peyrand advises that in order to obtain the full effects of the bromide the surface should be gently washed off just previous to the sprinkling of the powder.—*Allg. Wien. Med. Zeit.*, Feb. 23, '75.

